# Supplementary material for: Diagnosis and Management of Functional Tic-Like Phenomena
Source: J Clin Med. 2022 Oct 31;11(21):6470. doi: 10.3390/jcm11216470 (PMC9656241; doi:10.3390/jcm11216470)
Supplement: Supplementary file 1 [file jcm-11-06470-s001.zip › jcm-1935964-supplementary.pdf]

Supplementary Materials

Table S1. Data for features of functional *vs.* typical tics.

Authors	Year	Type of Feature	Feature	Evidence Type	Statistics for Feature of Interest	Comment
Buts et al.	2022	course	abrupt onset	34 consecutive youth with sudden-onset tic-like movements	34/34	defining characteristic for group; [aa]
Dreissen et al.	2016	course	abrupt onset	clinical experience, review		
Ganos et al.	2014	course	abrupt onset	case report		
Janik et al.	2014	course	abrupt onset	5 of 268 consecutive tic patients	5/5	
Mink	2013	course	abrupt onset	19 contemporary patients in the same small town	19/19	
Müller-Vahl et al.	2020	course	abrupt onset	clinical experience		
Paulus et al.	2021	course	abrupt onset	13 patients with symptoms after watching "Tourette" videos on social media, and 13 matched TS controls	12/12 (only in the FND sample) vs 0/13 with TS, p<.001	Meaning that "the time from symptom onset to ... impair[ment] to such a degree that ... medical advice was required was short, typically less than a day." Probably lower accuracy in young children
Pringsheim and Martino	2021	course	abrupt onset	prospective cohort of adults with tic-like phenomenology, 9 rapid-onset, 24 TS / chronic tic disorder	100% onset over a period of hours to days	
Pringsheim et al.	2021	course	abrupt onset	clinical experience at 8 centers across the world		
Hull and Parnes	2021	course	abrupt onset, recalling exact onset date	6 teenage girls seen in 3 months with abrupt-onset tic-like movements	5/6	
Hull and Parnes	2021	course	anatomic distribution: hand tics at initial presentation	6 teenage girls seen in 3 months with abrupt-onset tic-like movements	6/6	
Ganos et al.	2014	course	complex behaviors at onset	case report		
Pringsheim et al.	2021	course	complex behaviors at onset	clinical experience at 8 centers across the world		
Paulus et al.	2021	course	complex movements at onset	13 patients with symptoms after watching "Tourette" videos on social media, and 13 matched TS controls	7/13 vs 0/13 TS controls, p=.083	
Hull and Parnes	2021	course	complex vocalizations at onset	6 teenage girls seen in 3 months with abrupt-onset tic-like movements	6/6	

Müller-Vahl et al.	2020	course	does not peak around age 10-12	clinical experience		
Hull and Parnes	2021	course	dystonic tics at initial presentation	6 teenage girls seen in 3 months with abrupt-onset tic-like movements	6/6	
Buts et al.	2022	course	lack of spontaneous symptom fluctuations	34 consecutive youth with sudden-onset tic-like movements	23/34 (68%) had no waxing or waning	[aa]
Demartini et al.	2015	course	lack of spontaneous symptom fluctuations	11 FND patients		
Dreissen et al.	2016	course	rapid deterioration to maximal symptom severity	clinical experience, review		
Monday and Jankovic	1993	course	spontaneous remissions lasting up to 10 hours	18 patients with "psychogenic myoclonus"		[gg]
Pringsheim et al.	2021	demographics	age at onset	prospective cohort of children with tic-like phenomenology, 20 rapid-onset, 270 primary tic disorder	mean 13.9 vs 6.4 years, p<.0001	
Han et al.	2022	demographics	age of onset	consecutive child patients, 22 with functional tics, 163 with TS/CTD	mean 13.8 vs 6.8 years, p<.0001	
Paulus et al.	2021	demographics	age of onset	13 patients with symptoms after watching "Tourette" videos on social media, and 13 matched TS controls	15.31 ± 2.96 years vs 5.15 ± 2.79 years, p<.001	
Anderson et al.	2022	demographics	female	prospective national sample of emergency department diagnoses in adults	During the pandemic, for "tic disorders, women … (34.1%) and people aged 18 to 24 years … (73.3%) had the greatest increases" in cases.	Adult NSSP publication by CDC examined ED notes for indications of a tic disorder including TS. Substantial increase "(25.8%) in tic disorder–related ED visit counts for women; and [high increase] in visit counts for … tic disorders (25.8%) among adults aged 18 to 24 years after a COVID-19 case peak compared with during a peak."
Baizabal-Carvallo and Jankovic	2014	demographics	female	9 FND patients vs. 273 TS patients	55.6% vs. 21.5%, p=.03	
Buts et al.	2022	demographics	female	34 consecutive youth with sudden-onset tic-like movements	32/34 (94%)	[aa]
Ganos et al.	2019	demographics	female	clinical experience		

Han et al.	2022	de-mographics	female	consecutive child patients, 22 with functional tics, 163 with TS/CTD	100% vs 28%, p<.0001	
Heintz et al	2013	de-mographics	female	26 adults with FND resembling tics or myoclonus	8/26, vs 3/16 in TS, p=.39	sex **not** significantly different
Larsh et al.	2022	de-mographics	female	89 new patients with FTD-tics vs 89 new patients with TS	83/89 vs 42/89, p<.0001	[bb]
McVige et al.	2012	de-mographics	female	teens in a small town	13/14	[cc]
Mink	2013	de-mographics	female	19 contemporary patients in the same small town	18/19	
Monday and Jankovic	1993	de-mographics	female	18 patients with "psychogenic myoclonus"	13/18	[gg]
Olvera et al.	2021	de-mographics	female	TikTok videos labeled as tic or Tourette	64.3% female, 17.6% male, and 14.3% identified as nonbinary	
Pringsheim and Martino	2021	de-mographics	female	prospective cohort of adults with tic-like phenomenology, 9 rapid-onset, 24 TS / chronic tic disorder	100% vs 25%, p<.0001	
Pringsheim et al.	2021	de-mographics	female	clinical experience at 8 centers across the world		
Pringsheim et al.	2021	de-mographics	female	prospective cohort of children with tic-like phenomenology, 20 rapid-onset, 270 primary tic disorder	95% vs 21%, p<.0001	
Radhakrishnan et al.	2022	de-mographics	female	prospective national sample of pediatric emergency department diagnoses	Dramatic spike in ER visits for tic disorders in 2021, almost all in girls "The proportion of ED visits … among adolescent females … for tic disorders approximately tripled during the pandemic." (See their figure 2B)	Child NSSP publication by CDC examined ED notes for indications of tic disorders including TS. Note that other psychiatric disorders also increased substantially in adolescent females.
Heintz et al	2013	de-mographics	older at presentation	26 adults with FND resembling tics or myoclonus	mean age 53.6 vs 33.4 years in TS, p<.001	

Pringsheim and Martino	2021	demographics	older at presentation	prospective cohort of adults with tic-like phenomenology, 9 rapid-onset, 24 TS / chronic tic disorder	15.3 vs 10.1 years, p<.001,	
Pringsheim et al.	2021	demographics	older at presentation	prospective cohort of children with tic-like phenomenology, 20 rapid-onset, 270 primary tic disorder	mean 14.3 vs 10.5 years, p<.0001	
Müller-Vahl et al.	2020	demographics	onset after childhood (e.g. after age 8)	clinical experience		
Janik et al.	2014	demographics	onset at age 17 or later	5 of 268 consecutive tic patients		note: 3 of 5 also had typical tics in childhood
Buts et al.	2022	demographics	onset in adolescence	34 consecutive youth with sudden-onset tic-like movements	mean onset at 13.7 years	[aa]
Dreissen et al.	2016	demographics	onset in adolescence or adulthood	clinical experience, review		
Pringsheim et al.	2021	demographics	onset in adolescence or adulthood	clinical experience at 8 centers across the world		
Baizabal-Carvallo and Jankovic	2014	demographics	onset in adulthood	9 FND patients vs. 273 TS patients	mean age at presentation 36.3 vs 18.7 years, p = .014	
Demartini et al.	2015	demographics	onset in adulthood	11 FND patients		
Ganos et al.	2019	demographics	onset in adulthood	clinical experience		
Heintz et al	2013	demographics	onset in adulthood	26 adults with FND resembling tics or myoclonus	mean age 46.6 vs 15.8 years in TS, p=.002	
Müller-Vahl et al.	2020	demographics	onset in adulthood	clinical experience		
Pringsheim and Martino	2021	demographics	onset in adulthood	prospective cohort of adults with tic-like phenomenology, 9 rapid-onset, 24 TS / chronic tic disorder		
Ganos et al.	2014	demographics	onset in late adolescence	case report		
Monday and Jankovic	1993	initiating factor	(mostly minor) trauma 3 to 49 days prior to onset	18 patients with "psychogenic myoclonus"	6/14	[gg]

Mink	2013	initiating factor	exposure to similar symptoms, including by social media	19 contemporary patients in the same small town		
Han et al.	2022	initiating factor	exposure to social media content involving tics	consecutive child patients, 22 with functional tics, 163 with TS/CTD	18.2% vs (not reported)	
Pringsheim et al.	2021	initiating factor	exposure to social media content involving tics	prospective cohort of children with tic-like phenomenology, 20 rapid-onset, 270 primary tic disorder	20/20	exposure in TS/CTD group not reported
Buts et al.	2022	initiating factor	exposure to social media videos labeled as tics	34 consecutive youth with sudden-onset tic-like movements		[aa]
Pringsheim and Martino	2021	initiating factor	exposure to social media with #Tics or #Tourettes	prospective cohort of adults with tic-like phenomenology, 9 rapid-onset, 24 TS / chronic tic disorder	9/9	exposure in TS/CTD group not formally assessed, but all had >10 years of typical TS/CTD
Hull and Parnes	2021	initiating factor	exposure to the same social media tic videos	6 teenage girls seen in 3 months with abrupt-onset tic-like movements	6/6	
McVige et al.	2012	initiating factor	history of prior tic disorder diagnosis	teens in a small town	2/19	[cc]
Hull and Parnes	2021	initiating factor	history of significant family trauma	6 teenage girls seen in 3 months with abrupt-onset tic-like movements	4/6	possibly 5/6, text unclear
Baizabal-Carvallo and Jankovic	2014	initiating factor	history of tics in childhood	9 FND patients vs. 273 TS patients	0/9	family history not reported in the TS sample
Pringsheim et al.	2021	initiating factor	marked psychosocial stressors	clinical experience at 8 centers across the world		
Ganos et al.	2019	initiating factor	onset following trauma	clinical experience		
Müller-Vahl et al.	2020	initiating factor	onset immediately after viewing the same symptoms	clinical experience		usually in videos on social media platforms
Buts et al.	2022	initiating factor	other people in the environment with tics or tic-like symptoms	34 consecutive youth with sudden-onset tic-like movements	19/34	[aa]
Mink	2013	initiating factor	pending litigation or compensation	clinical experience		

Dreissen et al.	2016	initiat-ing factor	precipitating physical event or psychologi-cal stressor	clinical experience, re-view		
Buts et al.	2022	initiat-ing factor	previous diag-nosis of tics	34 consecutive youth with sudden-onset tic-like movements	15/34 (44%)	[aa]
Kurlan et al.	1992	initiat-ing factor	secondary gain	case report		"Clear" secondary gain
Müller-Vahl et al.	2020	initiat-ing factor	secondary gain	expert opinion		"All our patients had a clear secondary gain" (Müller-Vahl, personal communication)
McVige et al.	2012	initiat-ing factor	significant life stressors	teens in a small town	10/14	[cc]
van der Salm et al.	2012	labora-tory finding	absent Bereitschaftsp otential before intentional wrist exten-sion	controlled case series	specificity 98% in their sample (1/14 Tourette, 17/29 psycho-genic, 0/5 myo-clonus, 0/25 healthy control)	authors note the finding needs replication
van der Salm et al.	2012	labora-tory finding	Bereitschaftsp otential before tic-like phe-nomena	controlled case series		A BP may occur with tics, though with shorter onset la-tency (< 1 s)
Dooley et al.	1994	modi-fying factor	abnormal movements only on re-quest or when patient knew she was being observed.	Two patients with TS who developed later phenomena felt to be functional	1/2	
Ganos et al.	2019	modi-fying factor	atypical re-sponse to anti-tic medication	clinical experience		
Paulus et al.	2021	modi-fying factor	context de-pendent	13 patients with symp-toms after watching "Tourette" videos on so-cial media, and 13 matched TS controls	10/12 vs 0/13, p<.001	
Dreissen et al.	2016	modi-fying factor	decrease or disappear when not ob-served	clinical experience, re-view		
Pringsheim et al.	2021	modi-fying factor	disability but no impair-ment with preferred ac-tivities	clinical experience at 8 centers across the world		
Ganos et al.	2014	modi-fying factor	distraction may stop tics	case report		distraction minimized tics whereas intentional suppres-sion did not affect tics

Janik et al.	2014	modifying factor	dramatic improvement in unexpected situations	5 of 268 consecutive tic patients		when lying down or while smoking
Hull and Parnes	2021	modifying factor	dramatic response to low-dose medication	6 teenage girls seen in 3 months with abrupt-onset tic-like movements	1/6	all symptoms gone after 4 nights of guanfacine 0.5mg
Dreissen et al.	2016	modifying factor	entrainment to externally supplied rhythm	clinical experience, review		
Kurlan et al.	1992	modifying factor	highly suggestible	case report		
Dreissen et al.	2016	modifying factor	increase with attention	clinical experience, review		
Paulus et al.	2021	modifying factor	increased symptoms around other people	13 patients with symptoms after watching "Tourette" videos on social media, and 13 matched TS controls	12/12 vs 0/8, p<.001	
Dreissen et al.	2016	modifying factor	less suppressible	clinical experience, review		
Pringsheim et al.	2021	modifying factor	less suppressible	clinical experience at 8 centers across the world		
Monday and Jankovic	1993	modifying factor	marked reduction in amplitude with distraction	18 patients with "psychogenic myoclonus"		[gg]
McVige et al.	2012	modifying factor	media attention	teens in a small town	"Notable exacerbation and prolongation of symptomatology was observed with increased media attention. …"	[cc]
Baizabal-Carvallo and Jankovic	2014	modifying factor	not suppressible	9 FND patients vs. 273 TS patients	8/9	
Buts et al.	2022	modifying factor	not suppressible	34 consecutive youth with sudden-onset tic-like movements	14/34 (41%)	[aa]
Demartini et al.	2015	modifying factor	not suppressible	11 FND patients	9/11	
Hull and Parnes	2021	modifying factor	not suppressible	6 teenage girls seen in 3 months with abrupt-onset tic-like movements	6/6	
Janik et al.	2014	modifying factor	not suppressible	5 of 268 consecutive tic patients	5/5	

Mink	2013	modifying factor	not suppressible	19 contemporary patients in the same small town	19/19	
Müller-Vahl et al.	2020	modifying factor	not suppressible	clinical experience		
Paulus et al.	2021	modifying factor	not suppressible	13 patients with symptoms after watching "Tourette" videos on social media, and 13 matched TS controls	suppression in 5/12 vs 11/11, p=.082	
Robinson and Hedderly	2016	modifying factor	not suppressible	12 children with TS and "tic attacks"		[dd]
Paulus et al.	2021	modifying factor	not worse when alone	13 patients with symptoms after watching "Tourette" videos on social media, and 13 matched TS controls	worse when alone in 0/12 vs 8/8, p<.001	
McVige et al.	2012	modifying factor	psychosocial stressors	teens in a small town	"Notable exacerbation and prolongation of symptomatology was observed with ... psychosocial stressors."	[cc]
Pringsheim and Martino	2021	modifying factor	suggestible	prospective cohort of adults with tic-like phenomenology, 9 rapid-onset, 24 TS / chronic tic disorder	9/9	
Pringsheim and Martino	2021	modifying factor	suppressible	prospective cohort of adults with tic-like phenomenology, 9 rapid-onset, 24 TS / chronic tic disorder	6/9	
Paulus et al.	2021	modifying factor	symptoms continued during examination	13 patients with symptoms after watching "Tourette" videos on social media, and 13 matched TS controls	13/13 vs 1/10, p<.001	
Paulus et al.	2021	modifying factor	symptoms worsened in the presence of others	13 patients with symptoms after watching "Tourette" videos on social media, and 13 matched TS controls	12/12 vs 0/8, p<.001	
Monday and Jankovic	1993	modifying factor	treatment: improvement with placebo	18 patients with "psychogenic myoclonus"		[gg]
Baizabal-Carvallo and Jankovic	2014	modifying factor	treatment: lack of response to typical anti-tic medications	9 FND patients vs. 273 TS patients		

Janik et al.	2014	modifying factor	treatment: lack of response to typical anti-tic medications	5 of 268 consecutive tic patients		
Dreissen et al.	2016	modifying factor	treatment: marked response to placebo	clinical experience, review		
Monday and Jankovic	1993	modifying factor	treatment: marked response to placebo with suggestion	18 patients with "psychogenic myoclonus"		not tested comparably in Tourette syndrome; [gg]
Kurlan et al.	1992	modifying factor	treatment: no response to dopamine antagonists at doses higher than those that well controlled the patient's typical tics	case report		
Dreissen et al.	2016	modifying factor	treatment: respond less well to behavior therapy	clinical experience		authors note the limited evidence base
Kurlan et al.	1992	modifying factor	treatment: response to a brief course of psychotherapy	case report		
Hull and Parnes	2021	modifying factor	unusual symptom triggers	6 teenage girls seen in 3 months with abrupt-onset tic-like movements	5/6	e.g. other people, lights, 7p.m. nightly, whistling sounds
Zea Vera et al.	2021	modifying factor	very strong influence by the environment	social media videos	over half	[ff]
Monday and Jankovic	1993	modifying factor	worsened by stress, anxiety or exposure to noise or light	18 patients with "psychogenic myoclonus"	"Movements increased with 'stress' in 15, anxiety in 14, and exposure to noise in six and to light in four."	[gg]
Robinson and Hedderly	2016	other clinical feature	"increased focus on the tics in attempts to control the movements"	12 children with TS and "tic attacks"	12/12	[ee]
Dreissen et al.	2016	other clinical feature	ADHD and OCD not common	clinical experience, review		

Pringsheim and Martino	2021	other clinical feature	ADHD diagnosis	prospective cohort of adults with tic-like phenomenology, 9 rapid-onset, 24 TS / chronic tic disorder	22% vs 25%, NS	
Pringsheim et al.	2021	other clinical feature	ADHD diagnosis	prospective cohort of children with tic-like phenomenology, 20 rapid-onset, 270 primary tic disorder	25% vs 44%, p=.09	
Buts et al.	2022	other clinical feature	anxiety disorder	34 consecutive youth with sudden-onset tic-like movements	68%	in 23, prior diagnosis reported by parents; diagnosis in 17; [aa]
Pringsheim et al.	2021	other clinical feature	anxiety disorder	prospective cohort of children with tic-like phenomenology, 20 rapid-onset, 270 primary tic disorder	75% vs 19%, p<.0001	still significant controlling for age and sex
Pringsheim et al.	2021	other clinical feature	anxiety more severe	prospective cohort of children with tic-like phenomenology, 20 rapid-onset, 270 primary tic disorder	p<.0001	
Han et al.	2022	other clinical feature	anxiety or depression	consecutive child patients, 22 with functional tics, 163 with TS/CTD	95% vs 41%, p<.0001	
Buts et al.	2022	other clinical feature	autism spectrum disorder	34 consecutive youth with sudden-onset tic-like movements	12% reported, 57% suspected; 50% diagnosed clinically in a subset of 14 patients	[aa]
Kurlan et al.	1992	other clinical feature	borderline personality disorder	case report		
Baizabal-Carvallo and Jankovic	2014	other clinical feature	coexistence with other FNDs	9 FND patients vs. 273 TS patients		
Demartini et al.	2015	other clinical feature	coexistence with other FNDs	11 FND patients		
Ganos et al.	2016	other clinical feature	coexistence with other FNDs	13 patients with unusual vocalizations	5/13 cases	
Ganos et al.	2019	other clinical feature	coexistence with other FNDs	clinical experience		
Hull and Parnes	2021	other clinical feature	coexistence with other FNDs	6 teenage girls seen in 3 months with abrupt-onset tic-like movements	4/6	
Janik et al.	2014	other clinical feature	coexistence with other FNDs	5 of 268 consecutive tic patients	in 1/5 patients	

Heintz et al.	2013	other clinical feature	cognitive complaints without abnormal cognitive tests	26 adults with FND resembling tics or myoclonus	Cognitive compaints in 50% with FND, vs 37.5% with TS and 9.1% in healthy control groups (p=.03)	TS group performed worse than FND or healthy groups on Stroop color test
Buts et al.	2022	other clinical feature	comorbid psychiatric disorders	34 consecutive youth with sudden-onset tic-like movements	91%	prior diagnosis reported by parents; includes neurodevelopmental disorders; [aa]
Larsh et al.	2022	other clinical feature	current treatment by psychiatrist	89 new patients with FTD-tics vs 89 new patients with TS	36/89 vs 15/89, p=.002	[bb]
Larsh et al.	2022	other clinical feature	current treatment by psychologist or therapist	89 new patients with FTD-tics vs 89 new patients with TS	54/89 vs 29/89, p=.009	[bb]
Pringsheim and Martino	2021	other clinical feature	depression	prospective cohort of adults with tic-like phenomenology, 9 rapid-onset, 24 TS / chronic tic disorder	p=.03 for diagnosis of depression	
Pringsheim et al.	2021	other clinical feature	depression diagnosis	prospective cohort of children with tic-like phenomenology, 20 rapid-onset, 270 primary tic disorder	p<.0001, 55% vs 4%	
Pringsheim et al.	2021	other clinical feature	depressive symptoms more severe	prospective cohort of children with tic-like phenomenology, 20 rapid-onset, 270 primary tic disorder	p<.0001	
Dreissen et al.	2016	other clinical feature	exaggerated tendon reflexes or startle reactions	clinical experience, review		
Monday and Jankovic	1993	other clinical feature	functional sensory loss or weakness	18 patients with "psychogenic myoclonus"		[gg]
Robinson and Hedderly	2016	other clinical feature	marked anxiety prior to and during the attacks	12 children with TS and "tic attacks"	12/12	[ee]
McVige et al.	2012	other clinical feature	migraines	teens in a small town	9/14	[cc]
Pringsheim and Martino	2021	other clinical feature	more psychiatric comorbidity	prospective cohort of adults with tic-like phenomenology, 9 rapid-onset, 24 TS / chronic tic disorder	p<.05 for all self-report mental health symptom measures	
McVige et al.	2012	other clinical feature	neurological examination "findings consistent with conversion disorder"	teens in a small town		[cc]

Baizabal-Carvallo and Jankovic	2014	other clinical feature	no family history of tics	9 FND patients vs. 273 TS patients	9/9	
Buts et al.	2022	other clinical feature	no family history of tics	34 consecutive youth with sudden-onset tic-like movements	27/34 (71%)	[aa]
Demartini et al.	2015	other clinical feature	no family history of tics	11 FND patients	11/11	
Dreissen et al.	2016	other clinical feature	no family history of tics	clinical experience, review		
Ganos et al.	2016	other clinical feature	no family history of tics	13 patients with unusual vocalizations	13/13	
Hull and Parnes	2021	other clinical feature	no family history of tics	6 teenage girls seen in 3 months with abrupt-onset tic-like movements	6/6	
Heintz et al.	2013	other clinical feature	non-credible cognitive performance on a symptom validity test	26 adults with FND resembling tics or myoclonus	scores about 1 SD below those of Tourette syndrome and healthy comparison groups (p=.01)	
Ganos et al.	2016	other clinical feature	OCD	13 patients with unusual vocalizations	1/13	
Pringsheim and Martino	2021	other clinical feature	OCD diagnosis	prospective cohort of adults with tic-like phenomenology, 9 rapid-onset, 24 TS / chronic tic disorder	0/9 (0%) vs 8/24 (33%), p=.07	but much higher scores on self-report O-C symptom severity measure, consistent with somatization
McVige et al.	2012	other clinical feature	other functional symptoms	teens in a small town	6/14 developed syncope and functional seizures	[cc]
Dreissen et al.	2016	other clinical feature	other functional symptoms, current or past	clinical experience, review		
Heintz et al	2013	other clinical feature	use of brain-active medication	26 adults with FND resembling tics or myoclonus	8/26, vs. 2/16 in TS, p=.013	
Pringsheim and Martino	2021	other clinical feature	worse quality of life score	prospective cohort of adults with tic-like phenomenology, 9 rapid-onset, 24 TS / chronic tic disorder	mean 90.4 vs 60.1, p=.001	
Demartini et al.	2015	phenomenology	"blocking tics"	11 FND patients		interference with voluntary action is an item on the YGTSS
Janik et al.	2014	phenomenology	absence of simple motor tics of the face and neck	5 of 268 consecutive tic patients		

Müller-Vahl et al.	2020	phenomenology	absence of simple tics	clinical experience		
Zea Vera et al.	2021	phenomenology	aggression	social media videos	19.1%	[ff]
Dreissen et al.	2016	phenomenology	anatomic distribution: axial more often than head and neck	clinical experience, review		
Monday and Jankovic	1993	phenomenology	anatomic distribution: migrating	18 patients with "psychogenic myoclonus"		tics come and go over longer time intervals; [gg]
Ganos et al.	2019	phenomenology	anatomic distribution: tics mainly involve arms or trunk	clinical experience		
Paulus et al.	2021	phenomenology	anatomical distribution: predominantly involving trunk/extremities	13 patients with symptoms after watching "Tourette" videos on social media, and 13 matched TS controls	10/13 vs 0/11, p=.003	
Janik et al.	2014	phenomenology	atypical, non-tic-like behavior	5 of 268 consecutive tic patients		attacks of deep breathing, parkinsonism, or mutism for several days
Zea Vera et al.	2021	phenomenology	atypical, non-tic-like behavior		Senior clinician impression median Likert ratings = 5 (interquartile range 4-5, where 1 = "All the tics are typical of TS" and 5= "None of the tics are typical of TS"	
Han et al.	2022	phenomenology	complex phrases	consecutive child patients, 22 with functional tics, 163 with TS/CTD	45% vs 0.6%, p<.0001	
Pringsheim and Martino	2021	phenomenology	complex upper extremity tics	prospective cohort of adults with tic-like phenomenology, 9 rapid-onset, 24 TS / chronic tic disorder	89% vs 13%, p<.0001	
Pringsheim et al.	2021	phenomenology	complex upper extremity tics	clinical experience at 8 centers across the world		
Paulus et al.	2021	phenomenology	complex vocalizations	13 patients with symptoms after watching "Tourette" videos on social media, and 13 matched TS controls	5/12 vs 2/13, p=NS	

Pringsheim and Martino	2021	phenomenology	complex vocalizations	prospective cohort of adults with tic-like phenomenology, 9 rapid-onset, 24 TS / chronic tic disorder	89% vs 8%, p<.0001	
Pringsheim et al.	2021	phenomenology	complex vocalizations with a wide range of odd words or phrases	clinical experience at 8 centers across the world		
Zea Vera et al.	2021	phenomenology	complex vocalizations with long phrases (>3 words)	social media videos	45.7%	[ff]
Paulus et al.	2021	phenomenology	complex: predominantly complex	13 patients with symptoms after watching "Tourette" videos on social media, and 13 matched TS controls	12/13 vs 0/13, p<.001	
Han et al.	2022	phenomenology	coprolalia	consecutive child patients, 22 with functional tics, 163 with TS/CTD	77% vs 10%, p<.0001	
Hull and Parnes	2021	phenomenology	coprolalia	6 teenage girls seen in 3 months with abrupt-onset tic-like movements	2/6	
Janik et al.	2014	phenomenology	coprolalia	5 of 268 consecutive tic patients	2/5	
Olvera et al.	2021	phenomenology	coprolalia	TikTok videos labeled as tic or Tourette	50%	"93% had coprolalia or copropraxia versus 8% to 14% in the literature"
Pringsheim and Martino	2021	phenomenology	coprolalia	prospective cohort of adults with tic-like phenomenology, 9 rapid-onset, 24 TS / chronic tic disorder	67% vs 4%, p=.0004	
Pringsheim et al.	2021	phenomenology	coprolalia	clinical experience at 8 centers across the world		
Zea Vera et al.	2021	phenomenology	coprolalia	social media videos	53.2%	[ff]; coprophenomena present in over half in social media videos, vs. ~10% in TS patient visits
Ganos et al.	2016	phenomenology	coprolalia is atypical: longer, more complex	13 patients with unusual vocalizations		
Ganos et al.	2016	phenomenology	coprolalia with atypically high number of different swear words	13 patients with unusual vocalizations		
Ganos et al.	2016	phenomenology	coprophenomena at onset	13 patients with unusual vocalizations	7/13	

Buts et al.	2022	phenomenology	coprophenomena or echophenomena	34 consecutive youth with sudden-onset tic-like movements	26/34 (77%)	[aa]
Demartini et al.	2015	phenomenology	coprophenomena or echophenomena	11 FND patients	0/11	authors felt these phenomena were much less common in FND-tics
Han et al.	2022	phenomenology	copropraxia	consecutive child patients, 22 with functional tics, 163 with TS/CTD	45% vs 2%, p<.0001	
Zea Vera et al.	2021	phenomenology	copropraxia	social media videos	20.2%	[ff]; coprophenomena present in over half in social media videos, vs. ~10% in TS patient visits
Paulus et al.	2021	phenomenology	echolalia	13 patients with symptoms after watching "Tourette" videos on social media, and 13 matched TS controls	5/10 vs 0/13, p=.112	
Paulus et al.	2021	phenomenology	echopraxia	13 patients with symptoms after watching "Tourette" videos on social media, and 13 matched TS controls	5/9 vs 0/13, p=.082	
Müller-Vahl et al.	2020	phenomenology	exclusively complex and unusual movements or vocalizations	clinical experience		
Dreissen et al.	2016	phenomenology	feel completely involuntary to patient, rather than an almost inevitable response to a compelling urge	clinical experience, review		
Monday and Jankovic	1993	phenomenology	fluctuation between synchronous and asynchronous pattern	18 patients with "psychogenic myoclonus"		[gg]
Paulus et al.	2021	phenomenology	goal-directed movements, e.g. directed at others	13 patients with symptoms after watching "Tourette" videos on social media, and 13 matched TS controls	10/13 vs 0/13, p=.002	
Müller-Vahl and Edwards	2021	phenomenology	incongruity between reported severity and function			
Ganos et al.	2014	phenomenology	interference with voluntary action is common	case report		

Ganos et al.	2019	phe-nome-nology	interference with volun-tary action is common	clinical experience		
Baizabal-Carvallo and Jankovic	2014	phe-nome-nology	lack of pre-monitory phe-nomena	9 FND patients vs. 273 TS patients	0/9 had pre-monitory phe-nomena	
Buts et al.	2022	phe-nome-nology	lack of pre-monitory phe-nomena	34 consecutive youth with sudden-onset tic-like movements	62% (21/34) had premonitory phenomena, vs 77% historical control	no qualitative data available to compare urge phenomenology; [aa]
Demartini et al.	2015	phe-nome-nology	lack of pre-monitory phe-nomena	11 FND patients	2/11 had pre-monitory phe-nomena	
Dreissen et al.	2016	phe-nome-nology	lack of pre-monitory phe-nomena	clinical experience, re-view		urges have been described in functional phenomena
Hull and Parnes	2021	phe-nome-nology	lack of pre-monitory phe-nomena	6 teenage girls seen in 3 months with abrupt-on-set tic-like movements	4/6	
Mink	2013	phe-nome-nology	lack of pre-monitory phe-nomena	19 contemporary pa-tients in the same small town		
Müller-Vahl et al.	2020	phe-nome-nology	lack of pre-monitory phe-nomena	clinical experience		
Janik et al.	2014	phe-nome-nology	lack of sponta-neous symp-tom fluctua-tions	5 of 268 consecutive tic patients		
Müller-Vahl et al.	2020	phe-nome-nology	lack of sponta-neous symp-tom fluctua-tions	clinical experience		
Paulus et al.	2021	phe-nome-nology	lack of sponta-neous symp-tom fluctua-tions	13 patients with symp-toms after watching "Tourette" videos on so-cial media, and 13 matched TS controls	fluctuations "over weeks/months" in 0/12 vs 13/13, p<.001	
Buts et al.	2022	phe-nome-nology	lack of the typical ros-trocaudal tic distribution	34 consecutive youth with sudden-onset tic-like movements	62% started in the head or neck, vs 90% historical con-trols	[aa]
Demartini et al.	2015	phe-nome-nology	lack of the typical ros-trocaudal tic distribution	11 FND patients	2/11 lacked this distribution	
Ganos et al.	2014	phe-nome-nology	lack of the typical ros-trocaudal tic distribution	case report		
Olvera et al.	2021	phe-nome-nology	lack of the typical ros-trocaudal tic distribution	TikTok videos labeled as tic or Tourette	85.70%	arm movements more common than facial tics

Pringsheim et al.	2021	phe-nome-nology	lack of the typical ros-trocaudal tic distribution	clinical experience at 8 centers across the world		
Dreissen et al.	2016	phe-nome-nology	less stereo-typed and more variable than tics	clinical experience, re-view		
Buts et al.	2022	phe-nome-nology	more im-paired global functioning	34 consecutive youth with sudden-onset tic-like movements	More impaired CGAS, median 45 (range 35-75), vs. histori-cal control of 59 ± 7.9 (mean ± SD)	CGAS = Children's Global As-sessment Scale; [aa]
Ganos et al.	2014	phe-nome-nology	most symp-toms were not stereotyped	case report		
Paulus et al.	2021	phe-nome-nology	mostly slow and tonic movements	13 patients with symp-toms after watching "Tourette" videos on so-cial media, and 13 matched TS controls	12/13 vs 0/13, p<.001	
Larsh et al.	2022	phe-nome-nology	movements cause pain	89 new patients with FTD-tics vs 89 new pa-tients with TS	63/89 vs 48/89, p=.25	[bb]
Mink	2013	phe-nome-nology	not stereo-typed	19 contemporary pa-tients in the same small town		
Dreissen et al.	2016	phe-nome-nology	patient less likely to at-tempt to hide or camouflage movements	clinical experience, re-view		
Paulus et al.	2021	phe-nome-nology	premonitory sensations	13 patients with symp-toms after watching "Tourette" videos on so-cial media, and 13 matched TS controls	10/12 vs 12/13, p=NS	
Pringsheim and Mar-tino	2021	phe-nome-nology	premonitory urges	prospective cohort of adults with tic-like phe-nomenology, 9 rapid-on-set, 24 TS / chronic tic disorder	9/9	
Kurlan et al.	1992	phe-nome-nology	resembled a functional sei-zure	case report		
Dreissen et al.	2016	phe-nome-nology	rhythmic	clinical experience, re-view		
Pringsheim and Mar-tino	2021	phe-nome-nology	same symp-toms as on so-cial media	prospective cohort of adults with tic-like phe-nomenology, 9 rapid-on-set, 24 TS / chronic tic disorder	"several"	

Müller-Vahl and Edwards	2021	phe-nome-nology	selective effect of abnormal movements on intentional ac-tions		"unwanted obligations can no longer be performed, while fa-vorite activities can be con-ducted without any re-strictions"
Hull and Parnes	2021	phe-nome-nology	self-injurious behavior	6 teenage girls seen in 3 months with abrupt-on-set tic-like movements	2/6	
Olvera et al.	2021	phe-nome-nology	self-injurious behavior	TikTok videos labeled as tic or Tourette	85.7%, vs 14% in literature	"generally caused significant in-jury, with many users showing bruises or abrasions and them-selves crying as a result of these injuries"
Zea Vera et al.	2021	phe-nome-nology	self-injurious behaviors	social media videos	27.7%	[ff]
Han et al.	2022	phe-nome-nology	self-injurious tic-like behav-iors	consecutive child pa-tients, 22 with functional tics, 163 with TS/CTD	50% vs 4%, p<.0001	
Pringsheim et al.	2021	phe-nome-nology	self-injurious tic-like behav-iors	clinical experience at 8 centers across the world		
Dreissen et al.	2016	phe-nome-nology	striking dis-ruption of vol-untary move-ment	clinical experience, re-view		
Olvera et al.	2021	phe-nome-nology	striking dis-ruption of vol-untary move-ment	TikTok videos labeled as tic or Tourette	"89.3% had movements that were dis-ruptive to ac-tivity versus 12% to 29% in the literature."	
Buts et al.	2022	phe-nome-nology	symptoms "al-most identi-cal" to those in social media videos	34 consecutive youth with sudden-onset tic-like movements	14/34 (41%)	[aa]
Dooley et al.	1994	phe-nome-nology	symptoms were not stere-otyped	2 patients with TS who developed later phenom-ena felt to be functional		movements were not stereo-typed from one instance to the next
Zea Vera et al.	2021	phe-nome-nology	throwing ob-jects	social media videos	22.3%	[ff]
Ganos et al.	2019	phe-nome-nology	typically very aware of their tics	clinical experience		many people with TS or tics in autism are aware of none or only some of their tics
Janik et al.	2014	phe-nome-nology	unchanging clinical pat-tern	5 of 268 consecutive tic patients	2/5	
Ganos et al.	2016	phe-nome-nology	unusual pre-monitory sen-sory phenom-ena	13 patients with unusual vocalizations	9/13	9 of 13 had premonitory sensa-tions, summarized in text as be-ing atypical, with examples of "a sudden energy pulse" and "generalized whole body pres-sure"

Ganos et al.	2019	phe-nome-nology	unusual symptoms like pain or nausea can accompany tic suppression	clinical experience		
Monday and Jankovic	1993	phe-nome-nology	variable amplitude and frequency	18 patients with "psychogenic myoclonus"		[gg]
Paulus et al.	2021	phe-nome-nology	varied repertoire	13 patients with symptoms after watching "Tourette" videos on social media, and 13 matched TS controls	13/13 vs 0/13, p<.001	
Ganos et al.	2014	phe-nome-nology	very complex actions	case report		
Kurlan et al.	1992	phe-nome-nology	very different from patient's past tics	case report		
Kurlan et al.	1992	phe-nome-nology	very different from typical Tourette phenomenology	case report		
Olvera et al.	2021	phe-nome-nology	very frequent tics	TikTok videos labeled as tic or Tourette	Average 29 tics per minute	
Janik et al.	2014	phe-nome-nology	vocalizations and complex motor activities	5 of 268 consecutive tic patients	vocalizations (4/5), complex non-tic movements (4 of 5)	
Olvera et al.	2021	sever-ity	disabling	TikTok videos labeled as tic or Tourette		"Almost all recorded TikTok tics were severe, causing significant disability."
Robinson and Hedderly	2016	sever-ity	disabling	12 children with TS and "tic attacks"		[dd]
Han et al.	2022	sever-ity	disabling (missing school)	consecutive child patients, 22 with functional tics, 163 with TS/CTD	56% vs 7%, p<.0001	
Larsh et al.	2022	sever-ity	emergency department visit	89 new patients with FTD-tics vs 89 new patients with TS	27/89 (30.3%) vs 10/89 (11.2%), p=.048	[bb]
Buts et al.	2022	sever-ity	high severity	34 consecutive youth with sudden-onset tic-like movements	YGTSS 62.6 ± 19 (mean ± SD), vs historical control 25	[aa]
Janik et al.	2014	sever-ity	high severity	5 of 268 consecutive tic patients	5/5	
Larsh et al.	2022	sever-ity	home schooled because of movements	89 new patients with FTD-tics vs 89 new patients with TS	12/89 vs 2/89, p=.027	[bb]
Olvera et al.	2021	sever-ity	hospital visit due to movements	TikTok videos labeled as tic or Tourette	39.3%	

Han et al.	2022	sever-ity	hospitaliza-tion or emer-gency depart-ment visits	consecutive child pa-tients, 22 with functional tics, 163 with TS/CTD	36% vs 2%, p<.0001	
Larsh et al.	2022	sever-ity	impairment rated by child	children age ≥10 from among 89 new patients with FTD-tics and 89 new patients with TS	mean scores from mini-CTIM-C 9.9 tic-related, 11.1 other, vs 8.6, 11.2; p=.15, .49	[bb]; mini-CTIM-C = mini-child TS impairment scale, child ver-sion
Larsh et al.	2022	sever-ity	impairment rated by par-ent	89 new patients with FTD-tics vs 89 new pa-tients with TS	mean scores from mini-CTIM-P 10.0 tic-related, 10.3 other, vs 8.6, 9.2; p=.14, .37	[bb]; mini-CTIM-P = mini-child TS impairment scale, parent version
Janik et al.	2014	sever-ity	maximal se-verity at onset	5 of 268 consecutive tic patients		
Pringsheim and Mar-tino	2021	sever-ity	more severe symptoms	prospective cohort of adults with tic-like phe-nomenology, 9 rapid-on-set, 24 TS / chronic tic disorder	p<.01 for YGTSS motor and vocal tic and impair-ment scores	
Larsh et al.	2022	sever-ity	movements injure the body	89 new patients with FTD-tics vs 89 new pa-tients with TS	42/89 vs 10/89, p<.001	[bb]
Olvera et al.	2021	sever-ity	prolonged bouts of se-vere tics	TikTok videos labeled as tic or Tourette	64.3%	severe movements lasting for minutes to hours
Hull and Parnes	2021	sever-ity	prolonged bouts of un-ceasing tics	6 teenage girls seen in 3 months with abrupt-on-set tic-like movements	6/6	
Robinson and Hedderly	2016	sever-ity	prolonged bouts of un-ceasing tics (15 minutes to hours)	12 children with TS and "tic attacks"		[dd]
Olvera et al.	2021	sever-ity	severe	TikTok videos labeled as tic or Tourette		"almost all recorded TikTok tics were severe"
Pringsheim et al.	2021	sever-ity	severe	prospective cohort of children with tic-like phenomenology, 20 rapid-onset, 270 primary tic disorder	p≤.0001 YGTSS total tic score mean 33.3 vs 18.4, impair-ment mean 28.6 vs 15.8	greater YGTSS score when con-trolling for age and sex, p<.0001
Robinson and Hedderly	2016	sever-ity	severe	12 children with TS and "tic attacks"		[dd]
Buts et al.	2022	sever-ity	severe at on-set: initial presentation to an emer-gency depart-ment	34 consecutive youth with sudden-onset tic-like movements	16/34 (47%)	44% received medication in the emergency department; [aa]

Pringsheim et al.	2021	sever-ity	very severe at onset, often presenting for emergency care	clinical experience at 8 centers across the world

Table 1 notes.
[aa] Overlap with Pringsheim et al 2021 prospective child registry
[bb] All diagnosed clinically at a single referral center in 2021; TS sample enriched to match sample for age
[cc] Overlaps with group reported by Mink, 2013
[dd] Sample defined by this feature (among others), but interpreted as having FND contribute to symptom maintenance
[ee] Sample selected for prolonged, severe, nonsuppressible bouts but interpreted as having FND contribute to symptom maintenance
[ff] Not directly compared to a TS sample
[gg] May include some tic-like movements: the case definition included "movements that were best categorized as myoclonic because they were sudden and brief," with at least one patient showing rebound of kicking movements after suppression.
